# Substance-Induced Psychoses: An Updated Literature Review

**DOI:** 10.3389/fpsyt.2021.694863

**Published:** 2021-12-23

**Authors:** Alessio Fiorentini, Filippo Cantù, Camilla Crisanti, Guido Cereda, Lucio Oldani, Paolo Brambilla

**Affiliations:** ^1^Department of Neurosciences and Mental Health, Fondazione Istituto di Ricerca e Cura a Carattere Scientifico (IRCCS) Ca' Granda Ospedale Maggiore Policlinico, University of Milan, Milan, Italy; ^2^Department of Pathophysiology and Transplantation, University of Milan, Milan, Italy

**Keywords:** drugs, substance, substance (ab)use, induced psychosis, review

## Abstract

**Background:** On the current psychopharmacological panorama, the variety of substances able to provoke an episode of acute psychosis is rapidly increasing. Such psychotic episodes are classified according to the major category of symptoms: positive, negative, or cognitive psychotic episodes. On one hand, the abuse of methamphetamines, cannabis, and cocaine plays a big role in increasing the incidence of episodes resembling a psychotic disorder. On the other hand, the progress in terms of pharmacodynamics knowledge has led to the synthesis of new drugs, such as cannabinoids and cathinone's, which have rapidly entered into the common pool of abusers' habits. Regarding these newly synthesized substances of abuse, further clinical studies are needed to understand their psychogenic properties. The topic of this review is complicated due to the frequent abuse of psychotomimetic drugs by patients affected by psychotic disorders, a fact that makes it extremely difficult to distinguish between an induced psychosis and a re-exacerbation of a previously diagnosed disorder.

**Methods:** The present narrative review summarizes results from clinical studies, thus investigating the psychotogenic properties of abused substances and the psychotic symptoms they can give rise to. It also discusses the association between substance abuse and psychosis, especially with regards to the differential diagnosis between a primary vs. a substance-induced psychotic disorder.

**Findings:** Our findings support the theory that psychosis due to substance abuse is commonly observed in clinical practice. The propensity to develop psychosis seems to be a function of the severity of use and addiction. Of note, from a phenomenological point of view, it is possible to identify some elements that may help clinicians involved in differential diagnoses between primary and substance-induced psychoses. There remains a striking paucity of information on the outcomes, treatments, and best practices of substance-induced psychotic episodes.

## Background

According to the state of the art of literature, a relationship between drug abuse and the onset of psychotic symptoms is strongly supported (1, 2). In fact, plenty of findings prove that illicit substances (i.e., cannabinoids, cocaine, amphetamines, and hallucinogens) have psychotomimetic properties (3, 4). That is, their use can induce transient psychotic symptoms due to acute intoxication, but also possibly leading to a syndrome directly resembling a primary psychotic disorder (5).

Furthermore, over the last decades, a vast range of new psychoactive substances has emerged: synthetic cannabinoids, cathinone derivatives, psychedelic phenethylamines, novel stimulants, synthetic opioids, tryptamine derivates, phencyclidine-like dissociatives, piperazines, and GABA A/B receptors agonists are steeply becoming more rampant among the drug abuse panorama (6).

A struggling clinical dilemma is how to clearly identify a substance-induced psychosis from a primary psychotic illness or a psychotic illness with comorbid substance use. This could possibly be a subtle conundrum and a chance for elucubration, yet it becomes greatly important when treating and choosing the best therapeutic strategy for patients.

The Diagnostic and Statistical Manual of Mental Disorder, Fifth Edition (7) defines the substance-induced psychotic disorder as a psychiatric disease featured by delusions and/or hallucination during or soon after substance intoxication or withdrawal (please see Table 1). Furthermore, the symptoms of a psychotic disorder that is not substance-induced is yet to be properly understood.

**Table 1:** Diagnostic criteria of substance-induced psychosis according to the DSM-5 (7).

**Disorder**	**Criteria**
Substance-induced psychosis	A. Presence of one or both of the following symptoms: •Delusions •Hallucinations B. There is evidence from the history, physical examination, or laboratory findings that either (1) or (2): •The symptoms in Criterion A developed during, or within a month of, substance intoxication or withdrawal •Medication used is etiologically related to the disturbance C. The disturbance is not more accounted for by a psychotic disorder that is not substance-induced. D. The disturbance does not occur exclusively during delirium. E. The disturbance causes clinically significant distress or impairment in social, occupational, or other important areas of functioning.
Primary psychotic diseases	This group includes: •Schizophrenia •Other psychotic diseases •Schizotypal personality disorder All the previous conditions must have one or more symptoms of the following: •Delusions •Hallucinations •Disorganized speech •Disorganized behavior •Negative symptoms
Psychotic illness with comorbid substance use	At least, one of the criteria defining a psychotic disease and all the criteria of a substance use disorder must be present: •A pattern of use that results in marked distress and/or impairment, with two or more of the following symptoms for 12 months. •Using the substance in larger amounts or over a longer period of time than intended •Unsuccessful attempts or persistent desire to reduce its use •Excessive time spent on obtaining, using, and/or recovering from the effects of the substance •A pervasive craving for the substance •Significant interference with roles at work, school, or home •Continued use despite recurrent social or interpersonal consequences •Reducing or giving up important activities due to the substance use •Substance use in situations in which it may be physically hazardous •Substance use despite recurrent or persistent physical or psychological consequences •Tolerance of the substance •Withdrawal from the substance

The occurrence of drug-induced psychosis seems to be related to several pathogenetic mechanisms: (a) higher levels of central dopamine, especially for hallucinogens or psychedelic substances, stimulants and cathinone derivates, (b) a cannabinoid CB1-receptor agonist, in particular for cannabis-related substances (c) 5HT2A-receptor agonist for hallucinogenic plants, latest phenethylamines and tryptamine derivates, (d) antagonist activity at NMDA receptors (n-methyl-D-aspartate receptors) in ketamine and methoxetamine and, lastly, (e) k-opioid receptor activation in *Salvia divinorum* and *Mitragyna speciosa* (8).

A previous review of our group showed that psychosis due to substance abuse is a common issue in clinical practice and that the propensity to develop psychosis seems to be associated with the severity of use and dependence (2). However, this topic is continuously changing, with new substances coming out every day. Thus, an update of the previous review is highly required to create a better understanding of substance induced psychosis.

In this review, the presence of associated psychotic symptoms and the differences in clinical presentation will be analyzed for each substance. The second aim of our review is, in this complex framework, to perform an update of our previous work (2), in order to better define what is new and outstanding with regards to recently abused drugs, hopefully leading to a better comprehension of this topic.

## Methods

For this review, a PubMed, Medline and PsychINFO search for articles regarding drug abuse as causal or trigger factor for psychosis was performed; relevant studies were by two authors (FC and CC), and controversies were resolved by confrontation with a third author (GC).

In our article search, we only considered articles published from 01/01/2000, in order to exclude excessively outdated works. Specifically, articles of potential interest were identified by using the following terms: (substance^*^ OR drug^*^ OR inhalant^*^ cannab^*^ OR THC OR cathinone OR stimulant^*^ OR cocaine OR amphetamine OR methamphetamine OR hallucinogen^*^ OR LSD OR lysergic OR entactogen^*^[All Fields]) AND (psycho^*^ OR hallucin^*^ OR delusion^*^ OR parano^*^ [All Fields]).

The selected papers were included in the reference list only if meeting all the inclusion criteria, which consist of being (I) published in a peer-reviewed journal, (II) conducted in humans; (III) written in English and (IV) being an original study or a case-report. The exclusion criteria consisted of: (I) studies focusing on psychoses due to other causes than substances; (II) reviews, commentaries and book chapters; (III) abstracts from conferences; (IV) studies in animal-models and *in vitro* studies. The pertinent results were then selected on the basis of their relevance to the topic areas covered by the authors, and then synthesized for reporting and discussion. Titles of articles focused only on a small geographical area (e.g., Martinique, France or small Chinese populations), with patients who assumed more than one drug and on population with psychiatric comorbidities were excluded. Furthermore, we decided to exclude studies on the development of psychosis in patient at high risk or at ultra-high risk of developing psychosis. In conclusion, we also excluded not pertinent articles (e.g., forensic articles, psychosocial and not psychiatric articles).

In the choice of articles of interest for out paper, we initially identified, after the removal of duplicates, a number of 157 articles. We then removed not pertinent works (i.e., *n* = 55), and discrepancies were resolved by authors confrontation; a further number of 12 papers by other sources was found, and a total number of 72 papers was collected.

Due to the extent of the topic studied, it was not possible to adopt a systematic approach to analyse the data collected and to conduct a statistical analysis to compare them. Indeed, the substances described present evident heterogeneous features, and, for this reason, a descriptive approach has been adopted to provide a broad yet thorough overview of this topic.

## Results

### Clinical Differences in Psychoses

Despite the effort in defining clear-cut criteria of substance-induced psychosis, the results of the present review shows a picture of the complex relationship between psychotic symptoms and the use and abuse of illicit drugs. Furthermore, in most cases, chronological criteria are not sufficient to prove a direct causal effect between the substance and psychosis. In fact, in patients who use drugs and develop a psychotic episode for the first time, the evidence that such psychotic symptoms are primary and independent from drugs requires their persistence during a period of sustained abstinence from psychoactive substances. Indeed, drug-induced psychoses are expected to be resolved during a period of abstinence (9). On the other hand, subjects affected by drug-induced psychosis were more likely to abuse more than one drug and seemed to also show long-term hallucination after drug interruption (9).

In their longitudinal study, Mauri and colleagues examined the diagnostic and clinical courses of patients who used drugs while experiencing early-phase psychoses, with the aim to focus on the initial distinction between primary psychosis with drug abuse and substance-induced psychosis. The results obtained showed that the patients with primary psychosis had an earlier age of onset compared to the ones with drug-induced psychosis, it also showed baseline higher scores in the item called “unusual content of thought” according to the Brief Psychiatric Rating Scale (BPRS).

In their systematic review, Wilson et al. (10) summarized results from six studies, assessing the differences, particularly concerning psychopathology between subjects with a diagnosis of substance-induced psychosis and subjects affected by primary psychosis. The findings did not reveal several consistent differences. However, they found that, compared to primary psychosis, subjects affected by drug-induced psychosis showed a weaker family history of psychotic disorders, a greater degree of insight, fewer positive and negative symptoms, more depressive symptoms, and more anxiety.

The other important issue is that subjects who presented psychotic symptoms after substance abuse seemed to have a higher risk of the development of a primary psychotic illness (11). In fact, recent studies provide evidences that the abovementioned group of subjects is more likely to develop a schizophrenia-spectrum disorder or a primary psychotic disorder (11, 12).

In this regard, Starzer et al. (13) carried out a longitudinal study in a cohort of 6,788 subjects who received a diagnosis of substance-induced psychosis, investigating the rate of conversion to schizophrenia and bipolar disorder as well as risk factors for conversion. The results obtained showed a strong association between substance-induced psychosis and the development of either bipolar or schizophrenia-spectrum disorder. Moreover, young age was associated with a higher risk of converting to schizophrenia. Finally, self-harm episodes after substance-induced psychosis seemed to be significantly linked to a higher risk of converting to either schizophrenia or bipolar disorder.

In the following paragraphs we will discuss each single drug and its evidence on psychotic episodes. A useful summary on main data is reported in Table 2.

**Table 2:** Summary of major mechanism of action, nature of evidence and prevalence of psychotic symptoms for each substance.

**Substance**	**Major mechanism of action**	**Nature of evidence**	**Prevalence of psychotic symptoms**
Cannabinoids	Partial agonist activity on CB1	Partial agonist activity on CB1	0.8–10% (14)
Synthetic cannabinoids	Agonist activity on CB1	Case reports	NA
Synthetic catinones	Increase of dopamine release and inhibition of the reuptake of monoamines (15–19).	Cross-sectional studies	NA
Cocaine	SERT, DAT, and NET block (20), antagonism of the 5-HT3 receptor (21), and block of sodium channels (22)	Cross-sectional studies	Across the lifespan: 60.0–86.5% (23, 24)
Methamphetamines	Increase DA concentration by reversing VMAT2 and DAT (25)	Cross-sectional studies	17–37.1% (26, 27)
Hallucinogens	Increase 5-HT concentration, through agonist or partial agonist activity on 5HT receptors (28)	Case reports	NA
Entactogens	Increase 5-HT concentration through the inhibition of SERT and by reversing SERT, NA concentration, through the inhibition of NET and by reversing NET and DA concentration through the inhibition of DAT (28, 29).	Cross-sectional studies	NA
Phencyclidine and ketamine-induced psychosis	Antagonist activity on NMDA receptors Agonist activity on D2	Cross-sectional studies, randomized placebo-controlled clinical trial	NA

*5-HT, 5-hydroxytryptamine receptors; CB1, cannabinoids receptor 1; D2, dopamine deceptor 2; DA, dopamine; NMDA, N-methyl-D-aspartate; NA, evidence lacking in current literature*.

### Cannabinoids

#### Introduction

In recent years, there has been a significant increase in interest in the relationship between cannabis use and psychosis, partly because of concerns related to the growing availability of cannabis and its potential risks to health and human functioning.

#### Epidemiology

Cannabis is the most widely used illicit substance in the world, with 6–7% of the population in Europe and 15.3% of the population in the USA using it each year (30). According to the European Monitoring Center for Drugs and Addiction, in 2019 22.2 million adults used cannabis. The lifetime use of this substance can reach 27% of the European population.

The prevalence of psychotic features varies greatly depending on the content of THC in the cannabis, with THC having a direct relation with psychotic symptoms. Interestingly, since 2009 the power of cannabis (the THC content) increased and in some cases duplicated, while the price remained stable. Therefore, defining the real incidence and prevalence of psychotic disorders following cannabis use is not easy, with studies showing a range of 0.87–10.60%.

#### Pharmacodynamics and Toxicology

The clinical effects of cannabis are caused by its psychotropic main ingredient, delta-9-tetrahydrocannabinol (Δ9-THC), which acts as a partial agonist of Cannabinoids Receptor 1 (CB1) (31), widely expressed in the CNS, unlike its twin receptor CB2, mostly expressed on peripheral nerve terminals, on T cells of the immune system, on macrophages and B cells, and in hematopoietic cells.

THC is responsible for cannabis adverse effects, such as cognitive deficits (32) and psychotic and anxiogenic effects (33).

#### Symptoms and Clinical Characteristics

The psychoactive effects of Δ9-THC include psychotic symptoms, such as paranoia and hallucination, negative symptoms, feelings of disinhibition or dreaminess, sensations of heightened awareness of music, sounds, and colors or tastes (34). Acute episodes of cannabis-induced psychosis can last from few days to months, with a duration that varies consistently across studies (35).

Although most studies confirm the abovementioned psychogenic effect of Δ9-THC, discrepancies exist, highlighting the need to determine the consistency and magnitude of this finding.

On the other hand, in recent years, there has been an increasing interest in the properties of cannabidiol (CBD), the second most present cannabinoid in the common cannabis, which does not seem to induce psychotic symptoms, yet somehow anxiolytic and antipsychotic effects (If there are no such papers this should be stated.

In line with this knowledge, in their randomized, double blind clinical trial, Morgan et al. (36) administered cannabinoids by inhalation to 48 cannabis users; they planned four sessions, THC (8 mg), THC (8 mg) + CBD (16 mg), CBD 16 mg and placebo. They found an increase in psychotomimetic symptoms following administration of THC alone and the combination of THC and CBD, especially negative, perceptual distortions, and cognitive disorganization. Additionally, lower frequency cannabis users showed a reduction in symptoms following CBD administration alone, compared to placebo. A similar finding was reported by Kleinloog et al. (37) in a randomized, placebo-controlled, clinical trial on male mild cannabis-users. Specifically, transient positive symptoms were seen after THC administration, as measured by the Positive and Negative Symptoms Scale (PANSS).

Such symptoms seem to be associated with transient neurobiological modification, as proposed by Morrison et al. (38). In an RCT, comparing i.v. THC or placebo under double-blind condition during EEG recordings, they found that THC can decrease theta coherence between bi-frontal brain regions compared to placebo and that the reduction in coherence was strongly associated with positive psychotic symptoms.

The neurobiological underpinning of cannabis-induced psychotic symptoms, is however still not completely clear. The most common hypothesis involves an imbalance in dopamine levels in the prefrontal cortex (39). In line with this hypothesis, many genes involved in monoamines metabolism can modulate the effect of acute cannabis administration. Bhattacharyya et al. (40) showed that psychotogenic effects of cannabis were moderated by two genes that code for proteins that influence dopamine metabolism (i.e. DAT1 3' UTR VNTR and AKT1 rs1130233). Similarly, also polymorphisms in the catechol-O-methyltransferase (COMT) gene seems to moderate the psychosis-inducing effect of cannabinoids (41), thus strengthening the theory that dopamine moderation can influence psychosis and psychotic-like behaviors.

However, brain imaging SPECT studies reported that THC did not lead to a significant increase in dopamine release even though the dose was sufficient for participants to experience psychotic symptoms, suggesting a non-central role for striatal DA in THC-elicited psychosis (42).

Interestingly, Radhakrishnan et al. (43) tested the hypothesis that inducing a GABA deficit in healthy subjects might increase cannabis psychotomimetic properties. In fact, pre-treatment with iomazenil (an antagonist of benzodiazepines on GABA receptors), followed by THC administration, exacerbated subjective and psychophysiological effects of THC in healthy subjects and induced a significantly more severe psychotic episode when compared to THC alone.

In the discussion regarding the detrimental effect of cannabis abuse, there is a need to discuss the difference between a transient psychotic state induced by acute intoxication, a stable psychotic condition, and an increase in the risk of developing further primary psychiatric disorders. In this regard, the longitudinal study carried out by Manrique-Garcia et al. (44), found that cannabis is a risk factor for the development of schizophrenia in a cohort of 50,087 Swedish men with a history of cannabis use in late adolescence, followed up for 35 years. However, the risk was reported to be associated with the frequency and the dosage of cannabis, with moderate users showing a declining risk over the years. Similarly, a study that followed up 705 subjects for 6–60 months showed that the cessation of cannabis use may be beneficial in terms of reducing the psychotic experiences and subsequent risk of psychiatric disturbances (45).

Regarding the development of psychosis in specific populations, Valmaggia et al. (46), prospectively assessed the influence of cannabis use on the transition to psychosis in a sample of 184 subjects at Ultra-High Risk for psychosis, with a follow-up of 2 years. The results showed that lifetime, frequent, early-onset, and continued use were all associated with a more probable transition to psychosis. In addition, transition to psychosis was higher among those who started using cannabis before the age of 15 and continued frequent use.

This is corroborated by brain functional imaging studies showing that repeated exposure to cannabis during adolescence may have detrimental effects on brain resting functional connectivity, intelligence, and cognitive function (47).

Therefore, cannabis seemed to have acute psychogenic effects and longitudinal studies showed that cannabis can increase the risk of developing psychosis, especially in subjects already at risk for psychiatric disturbances. Moreover, cannabis seems to have the highest conversion rate (47%) to Schizophrenia compared to other substance-induced psychosis (13). However, such conversion seems associated also with the duration of cannabis use, since Shah et al. found that patients who completely abstained from cannabis after the 1st episode of Cannabis-Induced psychosis had no relapse of psychiatric illness (35). Moreover, the symptoms of presentation might have a predictive role for the consequent psychiatric disorder. In a recent study (35) half the patients who developed non-affective psychosis progressed to an independent psychotic disorder (e.g., schizophrenia), while only 7.7% of patients who developed predominantly affective psychosis developed an independent disorder. This latter result shows the importance of distinguishing non-affective from affective cannabis-induced psychosis in clinical practice. However, only a few studies have investigated the long-time effect and the clinical implications in the general population.

### Synthetic Cannabinoids

#### Introduction

Synthetic cannabinoids (SC), also known as Spice, K2, and Kush, are illicit narcotic substances commonly abused in United States, Europe, and Australia. They are composed of herbal mixtures, sprayed with various SCs (48).

#### Epidemiology

These substances have been available in Europe since 2004 (49) and are assumed typically by smoking or inhalation, similarly to cannabis itself; however, unlike cannabis, SCs are not detected by common drug screens (50).

Since the sixties, several synthetic compounds active on CBD receptors have been synthesized, introducing slight modifications to the original structure, thus, resulting in the emergence of three consecutive generations of synthetic cannabinoids (51).

#### Pharmacodynamics and Toxicology

Regarding pharmacodynamics, SCs mimic the action of Δ9-tetrahydrocannabinol (Δ9-THC), activating thus CB-1 receptors, resulting in psychotomimetic effects.

While on the one hand Δ9 THC is a partial agonist, SCs are full agonists, also with a higher affinity than Δ9 THC (48). Regarding the adverse effects of SCs (52), conducted a systematic review of case reports and case series on adverse events of synthetic cannabinoids, reporting in particular nausea, vomiting, pulmonary injuries, acute kidney injury, generalized tonic-clonic seizures, cardiovascular events and psychiatric conditions (i.e., psychosis, anxiety, paranoia, hallucinations).

#### Symptoms and Clinical Characteristics

In the last decades, several studies investigated the correlation between THC and psychiatric symptoms, in particular psychotic ones, but reports regarding SCs-induced symptoms are scattered and sporadic.

In subjects with a negative psychiatric history, case reports showed transient, acute, psychotic symptoms, such as paranoia, visual and auditory hallucinations, after smoking K2 or Spice (53–61).

Case reports about the occurrence of visual hallucinations after a single dose of JWH-018 (62) and the presence of JWH-018 metabolites from three people with paranoia and hallucinations (63) are also documented in the literature.

The risk to develop severe psychotic symptoms, however, seems to increase in presence of a previous psychiatric diagnosis. In this regard, two patients affected by Attention Deficit Hyperactivity Disorder (ADHD) showed a severe psychotic picture, in one case characterized by paranoid delusions and self-mutilation after an acute use of SCs (64), while in the other by catatonia, self-talk, and inappropriate laughter following 18 months of continuous and heavy SCs use (65).

Moreover, Peglow et al. observed a patient affected by post-traumatic stress disorder (PTSD) who had visual hallucinations and disorganized, bizarre behavior after Spice use (58).

Lastly, Rahmani et al. (66) described psychotic symptoms after using SCs in two adolescent males with a family history of schizophrenia, alcoholism, depression, and anxiety.

Moreover, in patients with a former diagnosis for psychiatric disorders and vulnerable, high-risk, individuals (67), SCs can precipitate the severity of the disturbances by eliciting a psychotic relapse. This seems particularly true for patients affected by schizophrenia and substance-induced psychosis. Specifically, Celofiga et al. (68) reported delusions and hallucinations after SCs use in patients with a history of schizophrenia. Similarly, in a follow-up study, Every-Palmer (69) reported that in 15 patients affected by schizophrenia, schizoaffective disorder, and bipolar affective disorder, 9 of them reported or exhibited psychotic symptoms after SCs use. Furthermore, patients with a previous history of substance-induced psychosis could present severe psychotic symptoms mainly with delusions after smoking Spice, and other SCs (61, 67, 70).

Overall, results obtained from the abovementioned case reports showed that SCs had an important psychogenic effect. However, further studies are needed to confirm these results. Particularly, longitudinal studies could better elucidate the SC's long-term psychogenic effects on abusers without psychiatric comorbidity and on subjects with psychiatric diagnoses.

### Cathinone Derivates

#### Introduction

Cathinone and its derivatives are drugs related to the family of phenethylamine (as amphetamines and methamphetamines), but with much lower potency (71).

Synthetic cathinones appeared in drug markets in 2005, when methylone, an analog of MDMA, was the first synthetic cathinone reported to the European Monitoring Centre on Drugs and Drug Addiction (EMCDDA). Even if it had been synthesized ~8 years earlier as an ephedrine homolog (72) the first reports of 4-methyl-methcathinone, also called mephedrone, emerged in Israel in 2007, spreading then in other countries to worldwide in a relatively short period (73), becoming the most used synthetic cathinone.

#### Epidemiology

Mephedrone is usually administered orally and snorted. Furthermore, being completely water-soluble, it can be injected intramuscularly or intravenously, or even taken by rectal administration using a needle-free syringe (74). When taken orally, mephedrone starts to give effects to consumers within 15–45 min after ingestion; when snorted the effects can be felt in minutes with a higher peak, usually reached after 30 min. In both oral and nasal use, the effects last for 2–3 h; Conversely, if taken intravenously, only half an hour (75).

#### Pharmacodynamics and Toxicology

As mephedrone use became more popular, many authors began to study the pharmacodynamics of this new psychoactive drug, finding, in particular, an increase of dopamine release and inhibition of the reuptake of monoamines (15–19).

Some authors administered mephedrone, MDMA, or amphetamine to rats, concluding that the first and the latter produced a rapid and steep increase of extracellular dopamine (496 and 412%, respectively), while for MDMA the percentage was significantly lower (235%). Parallelly, extracellular serotonin increased by 941% in the case of mephedrone, 911% in MDMA, and only 154% in amphetamine. Psychomotor excitability was the highest in amphetamine, and three times lower in the other two drugs studied.

Furthermore, according to the results of this study, the authors found that high doses of mephedrone cause a fast release of dopamine and serotonin from the nucleus accumbens, but conversely, the elimination rate of dopamine was as fast as the one of amphetamine, leading to a more potent and robust stimulation of the addiction circuits (76).

#### Symptoms and Clinical Characteristics

The main purpose and desired effects of mephedrone are caused by its entactogenic properties: increased subjective social skills, libido abilities, and a deep sense of wellness. The other more common side effects of mephedrone are sweating, headache, tachycardia, palpitations, nausea, thoracic pain, bruxism, psychomotor agitation, and paranoia. Whereas, cardiac, psychiatric, and neurological signs are some of the adverse effects reported by synthetic cathinone users, agitation, ranging from mild agitation to severe psychosis, is the most common symptom identified from medical observations (77); such symptom usually presents itself with aggressiveness, hallucinations, delusions, hyperactivity and, in rare cases, sudden death: this clinical syndrome is called “excited delirium,” also common in cocaine and amphetamines consumption (78, 79).

To date, no study reports a rate of development of an induced psychotic episode or exacerbation of psychotic symptoms in people already diagnosed.

### Cocaine

#### Introduction

Cocaine, also known as coke, is a stimulant used as a recreational drug. For thousands of years, indigenous people of South America have chewed the leaves of a plant (i.e., Erythroxylon coca) which is packed with plenty of alkaloids, including cocaine, isolated only in 1855 by Friedrich Gaedcke, who called such compound “erythroxyline” (80).

#### Epidemiology

It is well-known that cocaine use is associated with various mental disorders (81, 82) and that its consumption may lead to both transient psychotic symptoms (23, 24, 83, 84) and a complete induced psychosis (85).

#### Pharmacodynamics and Toxicology

Cocaine has been demonstrated to bind to the DAT transporter on the outward-facing conformation, blocking it in this conformation; in addition, cocaine also acts as an inhibitor of serotonin and noradrenaline reuptake, thus making cocaine a full-acting monoamine-reuptake inhibitor (20). Cocaine has also been shown to antagonize the 5-HT3 receptor, but the exact effects of its properties are still unclear (21). Cocaine also blocks sodium channels, interfering with the propagation of action potentials, making it act as a local anesthetic (22).

#### Symptoms and Clinical Characteristics

The onset of any psychotic feature during cocaine use is common, ranging from 29 to 86.5% (86), although these symptoms do not always accompany the intake and vanish with withdrawal (87–90).

Regarding the clinical features of psychotic symptoms in cocaine users, in a study on 55 patients with cocaine addiction, 29 reported psychotic symptoms: 90% of subjects had paranoid delusions, 96% experienced hallucinations (the most common were auditory), and 29% developed behavioral abnormalities (83).

In a larger European study, in which 173 patients were enrolled, 53.8% reported psychotic symptoms, with a neat higher frequency of paranoid and suspiciousness beliefs; hallucinations were also present, and data from previous studies confirmed the higher likelihood of visual ones (23).

Interesting data was found regarding the risk factors for developing psychosis during cocaine use. Firstly, the quantity of cocaine consumed is positively related to the onset of psychosis, with a significant correlation between dose and severity of symptoms (91). Additionally, using cocaine from a young age leads to higher vulnerability (83, 92–94), with a negative correlation between age at cocaine use onset and symptoms severity (87). Even psychiatric comorbidities were proven to be a risk factor, especially regarding ADHD (86), previous psychotic episodes (95), and various personality disorders (96), amongst which the most common are antisocial and borderline personality disorders.

The importance of genetics became fundamental in the study of risk factors: significant correlations for variants of the dopamine transporter (DAT) gene (97), the catechol-O-methyltransferase (COMT) gene, and other genes coding for enzymes implied in dopamine metabolism were found. Such results need further studies to be confirmed, as different studies have failed to prove significant different genotypes between patients with or without cocaine-induced psychosis (98–100).

### Methamphetamines

#### Introduction

The parent compound of this class of substances, amphetamine (contracted from alpha-methylphenethylamine), has been chemically modified leading to plenty of variants, including methamphetamine (METH; N-methyl-alpha-methylphenethylamine), methylenedioxyamphetamine (MDA) derivatives, and many others. Some amphetamines, including dextroamphetamine, methamphetamine, and the related methylphenidate, are widely used in the treatment of attention deficit hyperactivity disorder (ADHD), obesity, and narcolepsy. Amphetamine and methamphetamine share similar characteristics, and thus they are generally called “amphetamines,” while MDA derivatives (i.e., 3,4-methylenedioxyamphetamine, MDA); 3,4-methylenedioxy-N-methylamphetamine, MDMA or ecstasy; 3,4-methylenedioxy-N-ethylamphetamine, MDEA) for their effects are called “empathogens” or “entactogens.”

#### Epidemiology

According to the World Drug Report 2019 (101) published by the United Nations Office on Drugs and Crime, in 2017 there were an estimated 28.9 million past-year users of amphetamine and methamphetamine (MA), corresponding to 0.6% of the global population aged 15–64, 15% lower than the previously estimated 34.2 million in 2016, with the form of the MA used varying considerably in different regions.

Research has shown that MA-induced psychotic disorder (MIP) is a prevalent health concern among methamphetamine recreational users. It is to note that Vallersnes et al. (102), in the attempt of estimating the frequency of psychosis for different recreational drugs, found a prevalence of psychosis of 14.7% for amphetamine and 11.3% for methamphetamine, while collecting data of cases with acute toxicity induced by recreational drugs accessing the Emergency Departments (EDs). A recent meta-analysis of Lecomte et al. (103) of 17 studies indicated that 36.5% of MA misusers have a history of MIP and these prevalence rates were higher when only those with methamphetamine use disorder (MUD) are considered (43.3%) and when the period of assessment was a lifetime (42.7%) rather than current (22.1%). Such results are consistent with our precedent review (2) and with Gan et al. (27) which found that, in a population of 1,430 participants with MUD, the incidents of MIP was 37.1% in the sample according to DSM-IV. Finally, Su et al. (26) in a cross-sectional study among 1,685 abstinent methamphetamine users in China found that 17.0% had MIP.

#### Pharmacodynamics and Toxicology

Amphetamine reverses both vesicular monoamine transporter 2 (VMAT2) and the dopamine transporter (DAT) (25) to effectively increase synaptic concentrations of dopamine (DA) in the striatum in the nigrostriatal pathway. Amphetamines cause an augmented DA release also in the mesolimbic and the mesocortical pathways from the ventral tegmental area (VTA) into the nucleus accumbens (NAc) and the pre-frontal cortex (PFC) (104). The DA overflow in the striatum leads to excessive glutamate release into the cortex which might, over time, cause damage to GABAergic cortical interneurons, through impairment of NMDA receptors (104). This process may lead to a dysregulation of glutamate pathways transmitted through the thalamocortical signals and might result in the presentation of psychotic symptoms because of the damage to the cortex (104).

#### Symptoms and Clinical Characteristics

In 2018 Arunogiri et al. (105) published the first comprehensive review on 20 studies conducted in 13 populations to examine correlates of psychosis among people who use amphetamine and methamphetamine (MA). They found evidence that greater odds of psychotic symptoms were associated with more frequent MA use, the quantity of MA used, greater severity of MA addiction, and polydrug use. These results were later confirmed by other Authors, and MIP was associated with: earlier onset of drug use (27, 106–108), longer duration of MA use (26), higher MA use dose (26, 27), greater severity of MA addiction (27, 107, 108), polydrug use (26) nature of MA use (crystal methamphetamine vs. other forms of methamphetamine) (109) and comorbid depression or anxiety symptoms (26, 27, 108). Examining the prospective relationship between the duration of MA use and psychotic symptoms, it was found that the risk of experiencing psychotic symptoms was higher during periods of MA use compared with no use and, as the duration of MA exposure increased, the odds of experiencing psychotic symptoms also increased, with a clear dose-response effect of continued MA use on the risk of psychotic symptoms (110). Nie et al. (106), conversely, reported a negative association between the development of psychotic symptoms and higher dose of MA use, suggesting protection through tolerance, while Lamyai et al. (107) stated that the amount of MA use measured by hair analysis was not related to the experience of MA psychosis.

The onset of psychotic symptoms in a patient treated with an amphetamine drug, benzedrine, was reported for the first time in 1938 (111). In 1958, Connell (112) described 42 cases of amphetamine psychosis seen at the Maudsley Hospital in London. In the late Seventies, observing that an acute administration of amphetamines produced an accurate phenocopy of schizophrenia, several authors theorized that amphetamine-induced psychosis could be used as a model of schizophrenia (113) and that its continuous use could itself cause its development (114). Later, some authors suggested that once the use of amphetamine had induced symptoms, recurrences could be caused not only by its reuse but also by non-specific psychological stressors without any further use, suggesting an evolution of a lasting vulnerability state in the brain during chronic amphetamines abuse (115, 116).

More recently, Voce et al. (117) performed a systematic review of 94 articles that examined the symptom profile in individuals identified as having MIP. The most reported symptoms across all study types were persecutory delusions (reported in 84% of studies), auditory (69%) and visual (65%) hallucinations, hostility (53%), depression (31%), and conceptual disorganization (36%); negative symptoms did not appear characteristic of MIP (6–19%). The same group, Voce et al. (118) tried to determine with a three-factor model whether a discrete negative symptom syndrome exists in the psychiatric profile of methamphetamine users. They stated that negative symptoms exist among people who use methamphetamine, yet unlike positive or affective symptoms, they were not correlated with current methamphetamine use or with familial risks for psychosis, but appeared to be related to polysubstance use. Similarities and differences in the clinical features of MIP vs. schizophrenia have been studied by Warne and colleagues. While there has been considerable overlap between MIP and schizophrenia, visual and tactile hallucinations appear more prevalent in acute MIP, while schizophrenia is associated with pronounced thought disorder and negative symptoms (119).

Among MA users, most who experience psychotic symptoms showed a “transient psychosis,” which is experienced exclusively when using methamphetamine and recedes after intoxication (120). Some studies reported that a minority of people (up to 25%) experienced a “persistent psychosis,” i.e., a more prolonged psychosis that persists after stopping use of the drug (>1 month after abstinence) (117, 121). Lecomte et al. (122) suggested that severe psychotic symptoms, the duration of meth use, and sustained symptoms of depression were the strongest prognosticators of persistent psychosis. McKetin et al. (123) in a longitudinal prospective cohort study of addicted methamphetamine users, focused on the finding that persistent psychotic symptoms were more specifically related to a family history of a primary psychotic disorder and suggested that such individuals may have a pre-existing vulnerability to psychosis, following Tsuang (124) and Chen et al. (125).

### Hallucinogens

#### Introduction

The term “hallucinogen” was introduced in 1954 by Hoffer, who noted that for the first time certain drugs reproduced psychotic-like symptoms in healthy subjects (126). More recently, the term “psychedelic,” literally meaning mind-manifesting, has also been employed in the scientific community, to underline the fact that the psychological state induced by hallucinogens is not necessarily defined by pathological features (127). Nowadays, classic hallucinogens are considered such substances as those with a psychopharmacological profile resembling the one of mescaline, psilocybin, and lysergic acid diethylamide (LSD) (128).

There are two main chemical classes of classic hallucinogens: tryptamines and phenethylamines. The tryptamines include dimethyltryptamine (DMT), psilocybin (4-phosphoryloxy-DMT), and lysergic acid diethylamide (LSD); the phenethylamines include mescaline and many synthetic hallucinogens such as DOM and DOI (128).

#### Epidemiology

It is to note that Vallersnes et al. (102) found a prevalence of psychosis of 20.9% for LSD and 18.8% for psilocybe mushrooms, between recreational drugs users presenting themselves in an Emergency Department. These results should be examined in the light of important limitations (data on previous psychiatric diagnoses not collected, no follow-up data, diagnosis made by ED clinician); in fact, it is reported that a search in 2003 for case reports of LSD-induced psychosis found only three reports in the previous 20 years (128). Dos Santos et al. (129) found that the psychotic episodes associated with ayahuasca, a natural hallucinogen, and DMT intake described in the eight case series/case reports examined were associated with several contributing factors, and not only ayahuasca or DMT intake (es. personal or family history of psychiatric disorders, concomitant use of other drugs).

#### Pharmacodynamics and Toxicology

This class of drugs exerts its main effects increasing 5-HT brain levels, through agonist or partial agonist activity on 5HT receptors. 5-HT2A receptors seem to be the most important hallucinogenic targets, since the observation that 5-HT2A antagonists, like ketanserin, blocks tryptamines-mediated hallucinogenic effects (130). Classical hallucinogens should be considered as potent modulators of cortex network activity through the increase of the 5-HT2A agonist activity in the medial prefrontal cortex, the reduction of the inhibitory activity by the thalamic reticular nucleus, the altered firing of raphe nucleus, and the augmented activity in the locus coeruleus (28).

#### Symptoms and Clinical Characteristics

The acute effects of classic hallucinogens are similar, differing in duration and intensity, which depend on the specific substance, the dose, and the way of administration (128). The altered state of consciousness (ASC), a term coined by Ludwig (131), caused by this class of drugs comprises several symptoms: sensory alterations (affecting in particular visual perception, but also auditory and tactile ones), audio-visual synesthesia's and altered experience of time (127). Perception changes represent the most characteristic symptoms and include alteration in the perception of shape, size, and color, and the illusion of movement, but also more complex scenes. Anyway, alterations of visual perception infrequently represent true hallucinations, since they can usually be distinguished from real perceptions, at least at moderate doses (127).

The psychological content and emotional quality of the experience are unpredictable but are probably influenced by the mental state of the person who takes the drug, the environment in which the effects are experienced, and by the dose and the specific drug that has been taken (132). Liecthi (133) recently reviewed the controlled clinical studies of LSD published in the past 25 years. Focusing on psychotic symptoms, he found that disordered cognition could be a more fundamental characteristic of LSD's effects than positive or negative mood. However, LSD use was not characterized by unpleasant psychosis-like symptoms but instead by an overall positive mood state in the greater part of subjects.

Focusing on the long-term consequence of hallucinogen use in a retrospective cross-sectional study, Krebs and Johansen (134) reported that there were no significant associations between lifetime use of psychedelics and increased rate of any of the mental health outcomes. Furthermore, Rucker et al. (135) found that “no cases of prolonged psychosis or hallucinogen persisting perception disorder have been reported in modern trials with psilocybin, ayahuasca or LSD,” consistently with what was previously reported.

### Entactogens

#### Introduction

This class of drugs, due to their tendency to enhance emotions and empathy, was originally named “empathogen” in the 1980's. The term “entactogen,” meaning “*producing a touching within*,” was later adopted in 1986 (136) to avoid the ambiguous nature and negative connotations of the term “empathogen.” The main compound of entactogen is represented by 3,4-methylenedioxymethamphetamine (MDMA), popularly known as ecstasy. Such substance was first developed in 1912 (137), used as an adjunct to psychotherapy treatment beginning in the 1970's (138), and became popular as a street drug in the eighties. Entactogens constitute a subgroup of the amphetamine-type stimulants and are composed of MDA derivatives including three compounds: 3,4-methylenedioxyamphetamine (MDA), 3,4-methylenedioxy-N-methylamphetamine (MDMA), and 3,4-methylenedioxy-N-ethylamphetamine (MDEA). The main difference between ecstasy-type stimulants and other amphetamines/methamphetamines is the presence of a methylenedioxy group attached to the amphetamine aromatic ring in the former compound.

#### Epidemiology

Its popularity as a substance of abuse has increased over time; indeed, according to the World Drug Report 2019 (101) published by the United Nations Office on Drugs and Crime, in 2017 there were an estimated 21.3 million past-year users of “ecstasy,” corresponding to 0.4% of the global population aged 15–64. Vallersnes et al. (102) found a prevalence of psychosis of 4.3 % (20/461) between MDMA users presenting themselves in an Emergency Department.

#### Pharmacodynamics and Toxicology

MDMA increases brain levels of monoamines such as serotonin (HT), dopamine (DA), and norepinephrine (NE), *via* complex mechanisms (29). They may inhibit 5-HT reuptake through the inhibition of serotonin transporter (SERT) activity. They may also stimulate 5-HT release reversing SERT action through trace amine-associated receptor (TAAR1) agonism, inhibiting vesicular monoamine transporter (VMAT2), and inhibiting the monoamine oxidases (MAO) enzymes (28). Entactogens also act on norepinephrine transporter (NET), increasing norepinephrine release, and, with less affinity, on dopamine transporter (DAT), increasing dopamine release (28).

#### Symptoms and Clinical Characteristics

As already reported, MDMA combines a psychostimulant effect with highly unusual changes in consciousness, leading to euphoria and an intense love for oneself and others. In some cases, MDMA consumption can lead to psychotic symptoms, but most of the papers describing such features consist of single-case reports or small case series (139).

Soar et al. found that 29% of cases showing psychiatric symptoms after MDMA consumption involved psychotics symptoms and suggested that MDMA could cause long-term neurotoxicity (139). Additionally, they found that 24% of the patients had a previously diagnosed psychiatric history and only 34% had a family psychiatric history. Landobaso et al. (140) presented one of the largest samples of patients (32 patients) with psychotic symptoms induced by MDMA. They reported that the symptoms were most often positive, such as delusions (96%), hallucinations (96%), and conceptual disorganization (96%). Yet, negative symptoms were also detected, such as depressive mood (90%) and blunted affect (81%). Rugani et al. (141) compared the psychopathological symptoms of psychotic patients with (*n* = 23) and without (*n* = 46) recent use of MDMA, during their first psychotic episode and hospitalization, reporting that psychotic patients with recent use of MDMA were characterized by a less blunted affect and more hostile behavior.

Moreover, several authors reported that paranoid delusions and visual hallucinations could persist even several days after MDMA consumption (142). After the examination of several case reports, McGuire (143) reported that MDMA use may be associated with chronic psychiatric symptoms, which persist long after the cessation of MDMA use, such as psychotic features, panic disorder, depression, and obsessive-compulsive symptoms; however, it was not possible to determine whether MDMA use was directly responsible or it was incidental. Hallucinogen-persisting perception disorder (HPPD), a rare condition linked to hallucinogenic drugs consumption, is rarely diagnosed, yet it has been formulated due to the persistency of psychotic symptoms in a case report of a 19-year-old male (144). In 2014 Litjens et al. (145) presented 31 HPPD cases that implicated MDMA as a causative agent for HPPD-like symptoms, alongside classical hallucinogens.

### Phencyclidine and Ketamine-Induced Psychosis

#### Introduction

Phencyclidine (PCP) and ketamine are uncompetitive N-methyl-D-aspartate (NMDA) receptor antagonists (2) and act as short-acting general anesthetics for both human and veterinary use (146). In particular, their pharmacodynamics is the cause of the positive symptoms, related to the increased dopamine level in the prefrontal cortex and explained by the affinity of ketamine and PCP to the dopamine receptor 2 (D2) (147). Furthermore, such drugs determine the inhibition of GABAergic interneurons in the prefrontal cortex and increase neuronal activity, leading to an excessive glutamate release in the glutamatergic neurons of the prefrontal cortex (147). Interestingly, ketamine has recently emerged as a potential treatment for major depressive disorder. A single dose of 0.5 mg kg – 1 of ketamine has been shown to have rapid and relatively potent antidepressant effects.

#### Epidemiology

The annual prevalence of ketamine recreational use and abuse ranges from 0.8 to 1.8% in young adults (148). Since the recreational use of ketamine was first reported in the 1970's, it has become one of the most frequently used drugs, especially among young people and clubbers (149).

#### Clinical Features

Both PCP and ketamine show psychotogenic effects, including hallucinations, delusions, illogical thinking, reduced speech and thoughts, disturbance of emotions and affect, withdrawal, decreased motivation, and dissociation (2, 147) with a more powerful psychotic response showed by PCP compared to ketamine (147).

Interestingly, the cognitive and behavioral effects of PCP and ketamine in animals and humans are strongly similar to the positive and negative symptoms of schizophrenia, suggesting that abnormalities in NMDA receptor function might contribute to the biology of schizophrenia (150, 151).

Furthermore, differently from what was observed in amphetamine-induced psychosis, PCP and ketamine seem to also cause negative symptoms, such as apathy, reduced speech, perseveration, and catatonic posturing. In this regard, the abovementioned inhibition of GABAergic interneuron in the prefrontal cortex by PCP and ketamine is considered the neurobiological explanation for the negative symptoms (147).

Cheng et al. administered the Positive and Negative Syndrome Scale (PANSS) to a sample composed of non-psychotic ketamine users, psychotic ketamine users, and subjects affected by schizophrenia. The groups of psychotic ketamine users exhibited significantly greater total PANSS score and subscale score compared to non-psychotic ones, while such scores in psychotic ketamine users and schizophrenic patients did not differ significantly.

Accordingly, in their clinical trial, Hoflich et al. (152) found an increased PANSS score after i.v. ketamine administration compared to placebo in healthy volunteers and a significant increase of cortico-thalamic connectivity of the somatosensory and temporal cortex. Interestingly, these alterations of thalamic connectivity in healthy volunteers are similar to those reported for patients with schizophrenia (CIT).

In the clinical trial carried out by Driesen et al. (153), healthy volunteers show an increased PANSS score after i.v. ketamine administration. Moreover, through an fMRI, they found that changes of global brain connectivity in certain region-specific areas predicted the occurrence of psychotic symptoms.

Nagels et al. (154) found that ketamine administration to healthy volunteers elicited statistically significant psychopathological effects as assessed by PANSS. In fact, participants experienced perceptual abnormalities and dissociative states with a range of psychotic symptoms, including difficulties in thinking as well as in reality appraisal.

In a recent meta-analysis (155), which assessed the association between ketamine and psychiatric symptoms in healthy subjects, Beck et al. found that exposure to ketamine was associated with a statistically significant increase in transient psychopathology for total, positive and negative symptoms as measured by PANSS, compared to placebo, confirming thus previous results.

In conclusion, PCP and ketamine may transiently induce schizophrenia-like positive, negative, and cognitive symptoms in healthy subjects, leading to a paradigm shift from dopaminergic to glutamatergic dysfunction in the pharmacological model of schizophrenia. Interestingly, apart from the mentioned psychotic symptoms, in the last year's ketamine was approved as an antidepressant for treatment-resistant depression (156). Interestingly, although the dosage in depression is similar to those that can induce psychotic symptoms, recent studies suggest that the basal pre-drug state of the organism influence the overall outcome. Specifically, ketamine effects on NMDA receptors in depressive brains can lead to amelioration or remission of symptoms, whereas healthy individuals develop psychotic features (157).

However, the majority of the studies assessed in this review described the effects of ketamine and PCP in healthy subjects and patients affected by schizophrenia: further studies are needed to evaluate the recreative use of ketamine and PCP and their clinical implications.

## Discussion

In the last 20 years, plenty of papers have focused on psychosis induced by cannabinoids, cocaine, and methamphetamines. As we have already described in the previous review (2), despite the important diffusion of entactogens (MDMA and related substances) and classic hallucinogens (mescaline, psilocybin, and LSD) especially among young subjects, few papers are present on such compounds. Consequently, although the possible association with psychotic symptomatology seems clear, the scientific community is far from being able to provide conclusive evidence on this topic.

As an update of the abovementioned work, the most important finding is the increased number and variety of new psychoactive drugs. In time, more and more potent substances have been created and spread in their use, with more severe effects and consequences for recent users in comparison to the past. In fact, abuse of new drugs (i.e., synthetic cannabinoids, synthetic cathinones) has widened the panorama of secondary psychoses. Specifically, synthetic cannabinoids lead to a similar clinical syndrome to the one caused by cannabinoids, increasing the risk of the onset and chronicization of a fully structured psychotic disorder. Conversely, synthetic cathinones lead to “delirium-like” symptomatology, also called “excited delirium,” with increased psychomotor activity, rage, and increase impulsivity, ranging from a mild episode to a severe one, with sparse psychotic symptoms.

However, distinguishing between substance-induced psychosis, primary psychotic illnesses, and psychotic illnesses with comorbid substance use remains a difficult challenge for clinicians, such as the management of these patients in clinical practice.

The majority of the novel recreational substances are not part of routine urine screening (6) thus leading to a meaningful difficulty in ruling out a substance-induced psychosis in the differential diagnosis, e.g., in the emergency department where patients often present themselves acutely.

On the other hand, even when the diagnosis of substance-induced psychosis is formulated and psychotic symptoms decrease after metabolization and excretion processes, patients are frequently lost during follow-up (11, 158). This specific event is an issue, given that the relationship between substance-induced psychosis and the development of several mental illnesses is well-established and a long-term follow-up period is needed to identify, prevent, and effectively treat further relapses (13). In fact, up to 32.2% of substance-induced psychoses may convert to either schizophrenia or bipolar disorder (13). The highest conversion rate was observed in cannabis users. However, except for the substances most studied in the literature (namely, cannabis, cocaine, and methamphetamines), only a few data are available on the persistence of psychosis after acute intoxication and withdrawal. Moreover, even when the diagnosis of substance-induced psychosis is formulated, the conversion rate varies a lot across substances and studies and is influenced by several factors, such as subsequent abstinence (35) and individual vulnerability (46). In addition, younger age at the onset of substances abuse plays a fundamental role in the risk of a more probable conversion to a severe condition.

Such findings must be contextualized in the psychosocial background of a progressively younger age of onset of drug use, to focus on prevention strategies rather than diagnosis and treatment in the next future (11).

## Author Contributions

AF and PB contributed to conceptualization and full-text writing. FC, CC, GC, and LO contributed to full-text writing and article selection. All authors read and approved the final manuscript.

## Funding

This work was funded by Fondazione IRCCS Ca' Granda Ospedale Maggiore Policlinico di Milano (270 R20 and RC 270-02).

## Conflict of Interest

The authors declare that the research was conducted in the absence of any commercial or financial relationships that could be construed as a potential conflict of interest.

## Publisher's Note

All claims expressed in this article are solely those of the authors and do not necessarily represent those of their affiliated organizations, or those of the publisher, the editors and the reviewers. Any product that may be evaluated in this article, or claim that may be made by its manufacturer, is not guaranteed or endorsed by the publisher.
